# Circulating Adipokines in Alcohol-Related Liver Disease and MetALD: A Systematic Review and Structured Narrative Synthesis

**DOI:** 10.3390/ijms27146509

**Published:** 2026-07-22

**Authors:** Krystian Mirowski, Barbara Balicka-Ślusarczyk, Lubomir Skladany, Juan Pablo Arab, Ivica Grgurevic, Michał Kukla

**Affiliations:** 1 Department of Toxicology and Environmental Diseases, Jagiellonian University Medical College, 30-688 Krakow, Poland; barbara.balicka-slusarczyk@uj.edu.pl; 2 HEGITO Liver & Transplant Unit, Department of Internal Medicine 2, Faculty of Medicine, Slovak Medical University, F. D. Roosevelt Hospital, 975 17 Banska Bystrica, Slovakia; 3 Division of Gastroenterology, Hepatology, and Nutrition, School of Medicine, Virginia Commonwealth University, Richmond, VA 23298, USA; juanpablo.arab@vcuhealth.org; 4 Stravitz-Sanyal Institute for Liver Disease and Metabolic Health, Virginia Commonwealth University, Richmond, VA 23298, USA; 5 Department of Gastroenterology, Hepatology and Clinical Nutrition, University Hospital Dubrava, 10000 Zagreb, Croatia; 6 University of Zagreb School of Medicine, Faculty of Pharmacy and Biochemistry, University of Zagreb, 10000 Zagreb, Croatia; 7 Department of Gastroenterology and Hepatology, Jagiellonian University Medical College, 30-688 Krakow, Poland; michal.kukla@uj.edu.pl

**Keywords:** alcohol-related liver disease, MetALD, adipokines, chemerin, retinol-binding protein 4, omentin-1, vaspin, visfatin, eNAMPT, phosphatidylethanol

## Abstract

Alcohol-related liver disease (ALD) and metabolic dysfunction and alcohol-related liver disease (MetALD) are increasingly recognised as biologically heterogeneous conditions, but circulating novel adipokines have not been systematically synthesised in this setting. We searched PubMed/MEDLINE, Embase, Web of Science, Scopus and Cochrane CENTRAL from inception to 31 December 2025 for studies measuring chemerin, visfatin/nicotinamide phosphoribosyltransferase (NAMPT), vaspin, omentin-1 or retinol-binding protein 4 (RBP-4) in adults with ALD or MetALD; data were synthesised narratively using Synthesis Without Meta-Analysis (SWiM). Grey literature and non-English databases were not searched, which may have led to incomplete retrieval of small single-centre studies. Five studies were included. Direct ALD evidence came mainly from three cross-sectional alcoholic cirrhosis cohorts, while one population cohort linked baseline RBP-4 to incident MetALD/ALD. RBP-4 showed a phase-dependent pattern, increasing before incident MetALD/ALD but decreasing in established cirrhosis with impaired synthetic function. Chemerin was reduced, omentin-1 was markedly elevated, vaspin was nonspecific and historical visfatin/NAMPT assays were difficult to interpret. Current evidence supports a hypothesis-generating three-axis framework: hepatic source failure, impaired hepatic clearance/portal-systemic shunting and alcohol-driven adipose-liver inflammation. The available evidence chiefly reflects chronic alcohol-related cirrhosis together with limited incident MetALD/ALD risk data, rather than the full ALD/MetALD spectrum. Prospective MetALD-stratified cohorts with isoform-specific assays and objective alcohol biomarkers are required.

## 1. Introduction

Alcohol-related liver disease (ALD) encompasses a histological spectrum ranging from alcohol-related steatosis and steatohepatitis to cirrhosis and hepatocellular carcinoma. It is the most common cause of advanced liver disease in Europe and a leading indication for liver transplantation [1]. Despite a rising global burden, ALD remains undertreated and underprioritised relative to non-alcoholic steatotic disease [2]. In the United States, the contemporary alcohol-associated cirrhosis burden has risen disproportionately in young women and in adults below 45 years of age [3].

In 2023, a multisociety Delphi consensus replaced “non-alcoholic fatty liver disease” with “metabolic dysfunction-associated steatotic liver disease” (MASLD) and introduced the new category of metabolic dysfunction and alcohol-related liver disease (MetALD), defined by the presence of at least one cardiometabolic risk factor plus alcohol consumption of 140–350 g/week in women or 210–420 g/week in men [4]. Subsequent validation in a Danish prospective cohort confirmed that the new framework substantially redistributes patients between MASLD, MetALD and ALD with distinct prognostic implications, and that among individuals with a history of excessive alcohol intake 98% had at least one cardiometabolic risk factor [5]. Population estimates from the United States (NHANES 2017–2020) place the prevalence of MetALD at approximately 2% and of ALD at 0.7% among adults [6]. A 2025 expert panel position statement emphasises that the MetALD construct is biologically heterogeneous and methodologically demanding, because heavy alcohol use itself can produce hypertension, hypertriglyceridaemia and hyperglycaemia, and because accurate ascertainment of recent and lifetime alcohol exposure is essential to distinguish ALD from MetALD [7]. Adipokine literature in steatotic liver disease therefore largely pre-dates an era in which ALD, MetALD and MASLD can be discriminated reliably, particularly given that objective alcohol biomarkers such as phosphatidylethanol (PEth) have been shown to quadruple MetALD diagnoses and triple ALD diagnoses when added to self-report [8], and that hair ethyl glucuronide detects harmful alcohol consumption in approximately 29% of patients with presumed non-alcoholic fatty liver disease [9].

Adipokines are bioactive peptides secreted predominantly by adipose tissue and, to a varying degree, by the liver itself. They modulate inflammation, insulin signalling, hepatic stellate cell activity and fibrogenesis, positioning them as potentially informative circulating biomarkers of liver injury [10,11]. In ALD, chronic ethanol exposure activates adipose triglyceride lipase, increases free fatty acid efflux and drives reverse triglyceride transport from adipose to liver, with concurrent alterations in adipokine secretion [12]. Adipose-tissue atrophy (“alcoholic adipopenia”) and skeletal muscle loss are markedly more severe in alcoholic than in viral cirrhosis and independently predict mortality [13], further altering the circulating adipokine pool independently of hepatic biology. The relationship between alcohol and metabolic dysfunction is supra-additive: low-to-moderate alcohol consumption in MASLD shows a dose-dependent supra-additive interaction with cardiometabolic risk factors for fibrosis [14], and moderate alcohol intake synergises with type 2 diabetes mellitus to produce advanced fibrosis even in cohorts traditionally classified as non-alcoholic [15].

Classical adipokines such as leptin and adiponectin have been extensively studied in cirrhosis of various aetiologies. In contrast, five more recently characterised adipokines-chemerin (RARRES2), visfatin/NAMPT (also termed PBEF; existing in intracellular [iNAMPT] and extracellular [eNAMPT] forms), vaspin (SERPINA12), omentin-1 (ITLN1) and retinol-binding protein 4 (RBP-4)-have received growing attention as biomarkers of metabolic liver disease, yet their role in ALD and MetALD has not been systematically synthesised. Their candidate biology is summarised in the comprehensive review of adipokines in liver cirrhosis by Buechler and colleagues [16]: chemerin is predominantly produced by hepatocytes [17] and falls in advanced cirrhosis [18,19]; RBP-4 is hepatically synthesised and renally cleared, declining with hepatic synthetic failure and rising with renal impairment [20,21]; omentin-1 is visceral fat-derived and partly hepatically extracted, accumulating with portal-systemic shunting [22]; vaspin is a serpin-family adipokine of uncertain hepatic relevance; and eNAMPT has recently emerged as a uniquely ALD-relevant signal because ethanol selectively stimulates its secretion from brown adipose tissue, driving hepatocyte ferroptosis through TLR4-dependent mechanisms [23].

We conducted a systematic review to synthesise all available evidence on circulating levels of these five adipokines in adults with ALD or MetALD, compared with healthy controls or other liver disease populations, and to evaluate their associations with disease severity. We propose that adipokines in ALD and MetALD should not be interpreted as a single diagnostic panel but as a phase-dependent readout of three distinct biological axes: (i) hepatic source failure, (ii) impaired hepatic clearance and portal-systemic shunting, and (iii) an alcohol-exposure and inflammatory adipose-liver axis. Within this framework, the same adipokine may behave as a biomarker of risk, a biomarker of disease phase, or a candidate mechanistic mediator depending on where the patient sits along the steatotic liver disease continuum and on the quality of alcohol-exposure phenotyping.

## 2. Methods

### 2.1. Protocol and Registration

**Figure 1:**
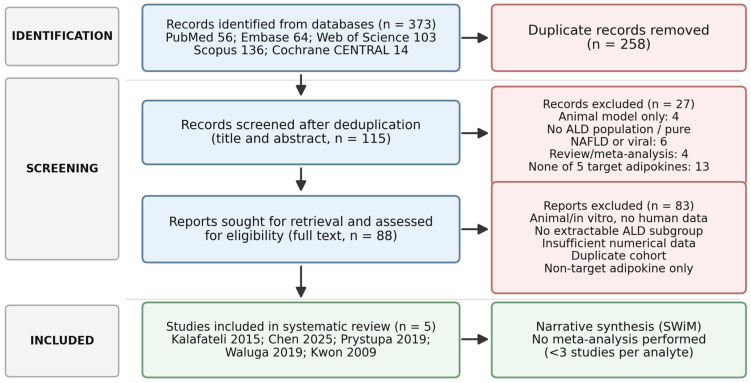
PRISMA 2020 flow diagram. The included studies are Chen 2025 [25], Kalafateli 2015 [26], Prystupa 2019 [27], Waluga 2019 [28] and Kwon 2009 [29]. PRISMA, Preferred Reporting Items for Systematic Reviews and Meta-Analyses; NAFLD, non-alcoholic fatty liver disease; ALD, alcohol-related liver disease; SWiM, Synthesis Without Meta-Analysis. The diagram was created by the authors using the Matplotlib library (version 3.10.8) for Python (version 3.12.3, Python Software Foundation, Beaverton, OR, USA).

This systematic review was conducted in accordance with the PRISMA 2020 guidelines and the SWiM reporting framework for narrative synthesis. The protocol was registered in the PROSPERO International Prospective Register of Systematic Reviews (registration number: CRD420261354251). The study selection process is summarised in the PRISMA 2020 flow diagram (Figure 1), and a completed PRISMA 2020 checklist is provided as Supplementary File S2 [24]. One minor amendment to the registered protocol was made after registration and is documented in the PROSPERO record (CRD420261354251); all other methods were conducted as described in the registered protocol.

### 2.2. Eligibility Criteria

Studies were eligible if they: (P) enrolled adults (aged at least 18 years) with ALD or MetALD, including mixed-aetiology cohorts in which ALD constituted at least 50% of participants or for which an ALD subgroup was reported separately; (I) measured circulating (serum or plasma) concentrations of at least one of the five target adipokines; (C) included a comparator group (healthy controls, other liver disease aetiology, or within-disease severity strata); and (O) reported numerical data (mean, standard deviation, median or interquartile range) sufficient for data extraction. A note on terminology: throughout this review we use the contemporary, non-stigmatising terms “alcohol-related” or “alcohol-associated” liver disease and cirrhosis in our own voice. The historical term “alcoholic cirrhosis” and the abbreviation “AC” are retained only where they reproduce the original terminology of an included study or its reported cohort labels; in those instances the term should be read as the historical designation used by the primary authors rather than as our preferred usage.

We included observational studies of all designs (prospective and retrospective cohort, cross-sectional, case-control). We excluded studies conducted exclusively in animal or in vitro models, review articles, editorials, letters without original data, studies reporting only tissue-level or mRNA expression without circulating measurements, and studies with fewer than ten participants per group. One retrospective mixed-aetiology study (Kwon 2009 [29]) was identified in which the alcoholic subgroup constituted 9.0% of participants and was not reported separately; this study does not meet the a priori 50% ALD threshold and was retained only as contextual evidence, not as primary ALD- or MetALD-specific evidence (see Section 3.1).

### 2.3. Search Strategy

A systematic search was performed in five databases: PubMed/MEDLINE, Embase (Ovid), Web of Science, Scopus and Cochrane CENTRAL (search date: 31 December 2025). The search combined three concept blocks: (1) ALD and chronic liver disease terminology, including the destigmatising shift from “alcoholic” to “alcohol-related/alcohol-associated” and the post-2023 SLD/MASLD/MetALD/ALD nomenclature; (2) the five target adipokines and their synonyms (chemerin/RARRES2/TIG2; visfatin/NAMPT/PBEF/eNAMPT/iNAMPT; vaspin/SERPINA12; omentin/omentin-1/ITLN1; RBP-4/retinol-binding protein 4); and (3) alcohol-related exposure terms. The full PubMed search string is provided in Supplementary File S1. Reference lists of included studies and relevant reviews were hand-searched. Formal grey literature retrieval (conference proceedings of the EASL International Liver Congress, AASLD The Liver Meeting and APASL Annual Meeting; preprint servers including medRxiv and bioRxiv; international trial and protocol registries beyond ClinicalTrials.gov; theses and institutional repositories) and native-language indices for major non-English literatures (eLibrary.ru, CNKI, SciELO, Polska Bibliografia Lekarska) were not searched; the implications of this restriction for retrieval bias and publication bias are addressed in Section 4.10.

### 2.4. Study Selection and Data Extraction

Records were imported into Zotero (version 9.0; Corporation for Digital Scholarship, Vienna, VA, USA) and deduplicated. Two reviewers (K.M., B.B-S.) independently screened titles and abstracts (Stage 1), followed by full-text review (Stage 2). Disagreements were resolved by discussion. Data were extracted using a pre-specified extraction form covering study design, country, sample size, patient characteristics (age, sex, body mass index, alcohol exposure, diabetes status), ALD diagnosis criteria, adipokine assay details (method, manufacturer, kit, units, isoform specificity where reported), circulating concentrations with measures of dispersion, between-group comparisons, and correlation coefficients. For studies with mixed-aetiology populations, only data from the ALD or alcohol-related subgroup were extracted when separately available.

### 2.5. Quality Assessment

Study quality was assessed independently by two reviewers using the Newcastle-Ottawa Scale (NOS) for observational studies. The NOS evaluates three domains: selection (maximum 4 stars), comparability (maximum 2 stars) and outcome (maximum 3 stars). Studies scoring at least 7 were classified as low risk of bias, 5–6 as moderate risk and 4 or fewer as high risk. Discrepancies were resolved by consensus. The two reviewers who performed the risk-of-bias scoring were K.M. and B.B.-S.; M. Kukla, a co-author of the present review and of Waluga 2019 [28], did not participate in the risk-of-bias assessment or the quality scoring of any included study, so the appraisal of Waluga 2019 was conducted independently of its co-author. We acknowledge that NOS is suboptimal for purely cross-sectional studies, in which reverse causality, selection of end-stage cases, and biomarker-related verification bias cannot be fully captured; to address this, we additionally appraised the three cross-sectional studies (Kalafateli 2015 [26], Prystupa 2019 [27] and Waluga 2019 [28]) using the JBI Critical Appraisal Checklist for Analytical Cross-Sectional Studies, which is better suited to capturing selection of end-stage cases and measurement bias in biomarker studies. The full item-level JBI appraisal is provided as Supplementary Materials (Supplementary Table S1 [30]) and did not alter the overall interpretation: the same relative ordering of study credibility was obtained, with Kwon 2009 [29] remaining the weakest source and the prospective cohort of Chen 2025 [25] the strongest. This limitation and the supplementary appraisal are further discussed in Section 4.10.

### 2.6. Synthesis

Because fewer than three independent studies were identified per analyte for the primary comparison of ALD versus healthy controls, quantitative meta-analysis was not performed. Results are presented as a structured narrative synthesis following the SWiM framework, grouped by adipokine. For each analyte, we report the direction and magnitude of differences, correlation coefficients with markers of disease severity, and diagnostic performance metrics where available. We organise the discussion around a three-axis biological framework intended as an integrating hypothesis (Section 4.1). Because no quantitative pooling was undertaken, the certainty of the body of evidence was not formally graded using the GRADE approach; instead, study-level risk of bias was appraised with the Newcastle-Ottawa Scale (Section 2.5), and the strength, consistency and limitations of the evidence base are evaluated narratively (Section 4.4, Section 4.5, Section 4.6, Section 4.7, Section 4.8, Section 4.9 and Section 4.10).

## 3. Results

### 3.1. Search Results and Study Characteristics

The database search yielded 373 records, of which 258 were duplicates. After screening 115 unique records at the title and abstract stage, 88 proceeded to full-text review. Five studies met all eligibility criteria and were included in the qualitative synthesis (Figure 1). The characteristics of included studies are summarised in Table 1. Before the individual findings are considered, one caveat should be kept in mind throughout: with the sole exception of the 2025 population cohort, all included studies were conducted and published before the 2023 multisociety nomenclature, and none applied the MetALD construct or objective alcohol biomarkers. Their populations were defined by legacy criteria for alcoholic cirrhosis, so the results below should be read as evidence pertaining predominantly to established alcohol-related cirrhosis rather than to the contemporary MetALD category.

The five included studies comprised one large prospective population-based cohort (Chen 2025, *n* = 3504 at baseline with 67 incident MetALD/ALD cases) [25], two cross-sectional studies enrolling only patients with alcoholic cirrhosis (Kalafateli 2015, *n* = 40 [26]; Prystupa 2019, *n* = 99 plus *n* = 20 healthy controls [27]), one cross-sectional study including an alcoholic cirrhosis subgroup within a three-disease cohort (Waluga 2019: *n* = 30 alcoholic cirrhosis, *n* = 25 controls [28]) and one retrospective study of mixed-aetiology chronic liver disease in which the alcoholic subgroup comprised 9.0% (*n* = 55/573) of the total (Kwon 2009 [29]). Studies were conducted in China, Greece, Poland (two studies) and South Korea. The adipokines examined were RBP-4 (three studies), chemerin (one study), omentin-1 and vaspin (one study each) and visfatin (one study). No study examined vaspin or visfatin as its primary analyte in a dedicated ALD or MetALD cohort.

The quantitative findings for individual adipokines are summarised in Table 2.

The nominal pooled sample of 4657 participants is dominated by the population-based baseline cohort of Chen 2025 [25] (n = 3504 free of SLD at inclusion), of whom 67 (1.9%) developed incident MetALD/ALD over 5 years. The cumulative directly informative ALD signal across cross-sectional studies of clinically diagnosed alcoholic cirrhosis is therefore approximately 169 cases (Kalafateli 2015 [26] plus Prystupa 2019 [27] plus Waluga 2019 [28]). Kwon 2009 is retained as contextual evidence only, because the alcoholic subgroup of 9.0% did not meet the a priori inclusion threshold and was not reported separately; we deliberately do not draw ALD- or MetALD-specific inferences from Kwon 2009 [29]. The present synthesis therefore comprises three layers of evidence: (i) direct evidence from alcohol-specific cirrhosis cohorts, (ii) one piece of contextual evidence from a mixed-aetiology cohort, and (iii) newer predictive evidence from a population-based MetALD/ALD reclassification cohort.

### 3.2. Risk of Bias Across Studies

NOS scores are reported in Table 3. Chen 2025 [25] achieved the highest score (9/9, low risk of bias), reflecting its prospective design, large sample size, and comprehensive adjustment for confounders including HOMA-IR and 12 metabolic covariates. Kalafateli 2015 [26] scored 7/9 (low risk), with strengths in patient selection (non-diabetic ALD-only cohort with at least 6 months of documented abstinence) and validated ELISA assays; points were lost for the absence of a healthy control group and the cross-sectional design. Prystupa 2019 and Waluga 2019 [27,28] were rated moderate risk (6/9 and 5/9, respectively), primarily due to insufficient adjustment for body mass index and metabolic confounders. Kwon 2009 [29] was rated high risk (4/9) because of its retrospective design, mixed-aetiology cohort without separate reporting for the alcoholic subgroup, and lack of comparability adjustment.

### 3.3. Retinol-Binding Protein 4 (RBP-4)

Two studies provided directly informative RBP-4 data in alcohol-specific or MetALD-specific populations, with one further mixed-aetiology study retained for context only.

In the Kalafateli 2015 [26] cross-sectional study of 40 non-diabetic patients with alcoholic cirrhosis (Child-Pugh A/B/C: 18/10/12), RBP-4 levels decreased progressively with worsening liver disease severity: Child-Pugh A 6.48 ± 3.2 microg/mL, Child-Pugh B 6.56 ± 3.37 microg/mL and Child-Pugh C 2.89 ± 2.07 microg/mL (*p* = 0.006). In multivariate linear regression, Child-Pugh stage was the only independent predictor of RBP-4 (standardised beta = −0.328, *p* = 0.04). RBP-4 was inversely correlated with MELD score (r = −0.439, *p* = 0.006), international normalised ratio (INR; r = −0.493, *p* = 0.008) and total bilirubin (r = −0.370, *p* = 0.029). Patients with ascites had significantly lower RBP-4 levels than non-ascitic patients (3.04 vs. 6.69 microg/mL, *p* = 0.001). No healthy control group was included [26].

In the prospective Chinese population-based cohort (Chen 2025) [25], 3504 participants without SLD at baseline were followed for 5 years, with 67 incident cases of MetALD/ALD (1.9%). Baseline RBP-4 levels were higher in participants who subsequently developed MetALD/ALD (median 62.0 mg/L, IQR 49.5–73.0) than in those who remained SLD-free (median 48.0 mg/L, IQR 39.0–60.0; *p* < 0.001). After adjustment for 12 covariates including age, body mass index, waist circumference, glycaemic indices, blood pressure, lipids and liver enzymes, a per-standard-deviation increment in RBP-4 was independently associated with MetALD/ALD incidence (relative risk 1.64, 95% CI 1.29–2.08, *p* < 0.001). The area under the ROC curve for RBP-4 in predicting MetALD/ALD was 0.672 in males, with an optimal cut-off of 59.00 mg/L.

The retrospective Korean study of Kwon 2009 [29] included a mixed-aetiology chronic liver disease cohort (HBV 61.9%, HCV 9.8%, alcohol 9.0%) and is retained here as contextual evidence only, because the alcoholic subgroup was not analytically separated and the alcoholic proportion did not meet the a priori 50% inclusion threshold. In that cohort, RBP-4 declined progressively across disease severity strata: normal controls 4.3 ± 1.1 mg/dL, chronic hepatitis 3.6 ± 2.0 mg/dL, Child A cirrhosis 2.6 ± 1.6 mg/dL and Child B/C cirrhosis 1.6 ± 1.0 mg/dL (*p* < 0.001 for all pairwise comparisons). The AUC for RBP-4 in diagnosing Child A cirrhosis was 0.717 (95% CI 0.669–0.766) and for Child B/C cirrhosis was 0.856 (95% CI 0.815–0.898). RBP-4 was most strongly correlated with prothrombin time (r = 0.541, *p* < 0.001) and serum albumin (r = 0.487, *p* < 0.001). Because the alcoholic subgroup was not reported separately, these data inform the general direction of RBP-4 with cirrhosis severity but cannot be used to support ALD- or MetALD-specific inference [29].

Taken together, the available evidence supports a phase-dependent pattern of RBP-4, with higher baseline levels in individuals who later develop MetALD/ALD and lower levels in established alcohol-related cirrhosis, where hepatic synthetic failure probably dominates the signal. Direct longitudinal demonstration of this trajectory within a single MetALD or ALD cohort is not yet available. A further interpretive caveat is that none of the three studies contributing RBP-4 data reported estimated glomerular filtration rate or serum creatinine, nor adjusted for renal function. Because RBP-4 is renally cleared and substantially elevated in chronic kidney disease independently of hepatic synthesis [20,21], unmeasured renal impairment-which is common in decompensated cirrhosis and hepatorenal syndrome-may confound the observed associations; therefore, future ALD/MetALD studies should routinely report renal function.

### 3.4. Chemerin

One study examined chemerin in an ALD-specific population. Prystupa 2019 [27] measured serum chemerin using a validated ELISA in 99 patients with alcoholic cirrhosis (Child-Pugh A: *n* = 29; B: *n* = 36; C: *n* = 34) and 20 healthy controls. Chemerin concentrations decreased progressively with advancing cirrhosis: controls 182.6 ± 80.4 ng/mL, Child-Pugh A 175.7 ± 62.7 ng/mL, Child-Pugh B 150.2 ± 59.7 ng/mL and Child-Pugh C 110.3 ± 73.6 ng/mL. Significant differences were observed between controls and Child-Pugh C patients (*p* = 0.01) and between Child-Pugh A and Child-Pugh C patients (*p* = 0.02).

Chemerin was inversely correlated with serum bilirubin (r = −0.48, *p* < 0.001), INR (r = −0.56, *p* < 0.001) and mean corpuscular volume (r = −0.36, *p* = 0.004), and positively correlated with albumin (r = 0.45, *p* = 0.003) and platelet count (r = 0.37, *p* = 0.003). In stepwise multiple linear regression, bilirubin (standardised beta = −0.42), albumin and platelet count were independent predictors of chemerin concentration, explaining 52% of its variance (adjusted R-squared = 0.52, *p* < 0.001). The AUC for chemerin in detecting liver cirrhosis was 0.653 (95% CI 0.50–0.81); the accompanying *p* = 0.51 denotes the test of the AUC against the null value of 0.5 (discrimination no better than chance) rather than any between-group concentration comparison, and these figures are reported exactly as published by Prystupa 2019 [27]. Chemerin therefore did not significantly discriminate cirrhosis from controls. At the optimal cut-off of 159 ng/mL, sensitivity was 64% and specificity 69%.

### 3.5. Omentin-1

One study reported omentin-1 concentrations in an ALD-containing cohort. Waluga 2019 [28] measured plasma omentin-1 using a validated BioVendor full-length-specific ELISA in 75 patients with chronic liver disease (NAFLD *n* = 25, primary biliary cholangitis *n* = 20, alcoholic cirrhosis *n* = 30) and 25 healthy controls. Plasma omentin-1 concentration was highest in the alcoholic cirrhosis group (median 1054.5, IQR 579.7–2208.9 ng/mL) compared with NAFLD (median 266.6, IQR 63.1–511.3 ng/mL), primary biliary cholangitis (median 408.8, IQR 207.4–808.2 ng/mL) and healthy controls (median 114.5, IQR 57.3–176.8 ng/mL; *p* < 0.01 for all comparisons with the alcoholic cirrhosis group).

Within the alcoholic cirrhosis subgroup, omentin-1 positively correlated with serum bilirubin (r = 0.502, *p* < 0.01) and plasma glucose (r = 0.390, *p* < 0.05), and negatively correlated with platelet count (r = −0.379, *p* < 0.05) and erythrocyte count (r = −0.451, *p* < 0.05). No significant correlation was found between omentin-1 and Child-Pugh score or MELD score. The authors attributed the markedly elevated omentin-1 levels (an approximately nine-fold excess over controls) to impaired hepatic metabolism of this adipokine, in keeping with the portal vein > systemic vein > hepatic vein omentin-1 gradient demonstrated by Eisinger and colleagues, which indicates first-pass hepatic extraction in cirrhosis [22]. Notably, the wider metabolic literature consistently shows that omentin-1 is reduced in obesity and insulin resistance; the markedly elevated values in alcohol-related cirrhosis therefore cannot plausibly be attributed to healthier adipose-tissue function and are consistent with a clearance/shunting signal. Waluga 2019 [28] did not measure the hepatic venous pressure gradient, so the relationship between omentin-1 and portal pressure could not be tested directly. The internal correlation structure of the dataset is nonetheless consistent with the clearance/shunting interpretation: the two significant associations reported-a positive correlation with bilirubin and an inverse correlation with platelet count-map respectively onto impaired hepatic excretory and metabolic function and onto thrombocytopenia, an established non-invasive surrogate of clinically significant portal hypertension, so that omentin-1 rose as this surrogate index of portal hypertension worsened. The concurrent absence of any correlation with Child-Pugh or MELD score, neither of which captures portal haemodynamics well, argues that omentin-1 indexes the clearance-shunting axis specifically rather than global hepatic failure. This evidence is indirect and confounded by overall disease severity, and the striking between-disease contrast is further confounded because only the alcohol-related group was explicitly defined as cirrhotic; a definitive test requires paired HVPG measurement. Within those limits, the available data are consistent with, but do not establish, the portal-systemic shunting hypothesis.

### 3.6. Vaspin

Vaspin concentrations were also reported in Waluga 2019 [28]. Vaspin concentrations did not differ significantly between the alcoholic cirrhosis group (median 0.27, IQR 0.093–0.84 ng/mL), NAFLD, primary biliary cholangitis and healthy controls (*p* = ns for all pairwise comparisons). Within the alcoholic cirrhosis subgroup, vaspin correlated positively with serum bilirubin (r = 0.411, *p* < 0.05) and negatively with erythrocyte count (r = −0.500, *p* < 0.01) and haematocrit (r = −0.475, *p* < 0.01), but showed no relationship with Child-Pugh score or MELD score.

### 3.7. Visfatin/NAMPT

Visfatin was measured in Kalafateli 2015 [26] using a Phoenix Europe ELISA. Serum visfatin levels did not differ significantly across Child-Pugh stages (Child-Pugh A: median 3.74 ng/mL, B: 12.32 ng/mL, C: 4.50 ng/mL; *p* = 0.536). After adjustment for fat mass, visfatin showed a numerically increasing trend with advancing cirrhosis stage, although this did not reach statistical significance in multivariate analysis. The isoform measured (eNAMPT vs. iNAMPT) was not specified by the authors. No study examined visfatin/NAMPT in a direct ALD-versus-control comparison with an adequate comparator group, and the historical visfatin data are largely uninterpretable in light of contemporary eNAMPT biology (see Section 4.2).

## 4. Discussion

This systematic review is the first to comprehensively synthesise evidence on five novel adipokines-RBP-4, chemerin, omentin-1, vaspin and visfatin/NAMPT-specifically in the context of ALD and the newly defined MetALD category. Our principal findings are: (1) RBP-4 shows a phase-dependent pattern, with elevated baseline values predicting incident MetALD/ALD in a population-based prospective cohort and reduced values in established alcohol-related cirrhosis that decline further with worsening hepatic synthetic function; (2) chemerin is decreased in alcohol-related cirrhosis and mirrors the decline in hepatic biosynthetic capacity; (3) omentin-1 is paradoxically elevated in alcohol-related cirrhosis compared with controls and with other chronic liver diseases; (4) vaspin levels are not significantly altered in ALD; (5) the available visfatin data are largely uninterpretable, particularly because no included study discriminated between the biologically distinct intracellular and extracellular isoforms; and (6) the cumulative direct ALD/MetALD signal across the included literature is substantially smaller than the nominal pooled sample size, and no analyte permits formal meta-analysis.

### 4.1. A Three-Axis Biological Framework for Adipokines in ALD and MetALD

**Figure 2:**
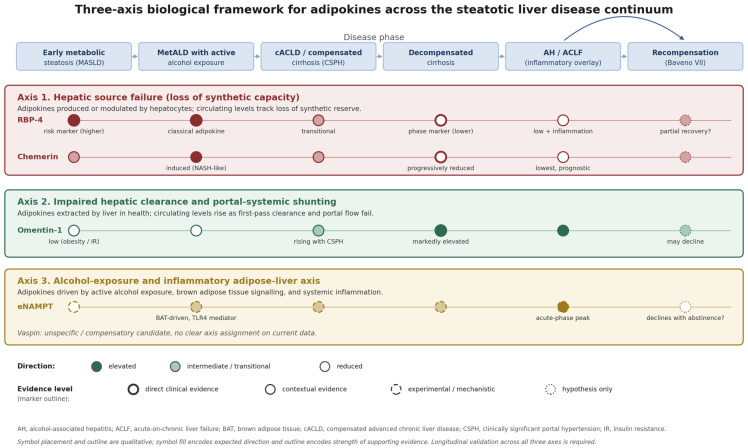
Three-axis biological framework for adipokines across the steatotic liver disease continuum.

We propose that the apparent contradictions in the published adipokine literature in steatotic liver disease cease to be noise once interpreted through a three-axis biological model that decouples adipokine signals into the dominant physiology they report (Figure 2).

Disease phase progresses from early metabolic steatosis (MASLD) through MetALD with active alcohol exposure into compensated advanced chronic liver disease (cACLD) and clinically significant portal hypertension (CSPH), decompensated cirrhosis, alcohol-associated hepatitis or acute-on-chronic liver failure (AH/ACLF, inflammatory overlay), and Baveno VII recompensation following sustained abstinence. The curved arrow indicates this recompensation pathway. The same adipokine signal carries a different biological meaning across this continuum, depending on which of three axes is dominant: (i) hepatic source failure (RBP-4, chemerin), (ii) impaired hepatic clearance and portal-systemic shunting (omentin-1), or (iii) the alcohol-exposure and inflammatory adipose-liver axis (eNAMPT). Vaspin remains unspecific on current data. Symbol strength additionally encodes the level of supporting evidence for each adipokine along each axis: solid symbols denote direct clinical evidence from an included study, half-filled symbols denote contextual evidence extrapolated from the wider cirrhosis literature, open symbols denote experimental or mechanistic evidence, and dotted outlines denote hypothesis only (see key). Symbol placement is qualitative and represents the dominant expected direction based on current evidence and biological inference.

This framework is a hypothesis, not a proven model. Symbol positions represent expected directions inferred from current evidence and biological reasoning, and longitudinal validation across all three axes is required. The figure was created by the authors using the Matplotlib library (version 3.10.8) for Python (version 3.12.3, Python Software Foundation).

The first axis is hepatic source failure. Adipokines that are either produced predominantly by hepatocytes or strongly dependent on hepatic synthetic capacity fall as functional liver mass is lost. Under this frame, the consistent reduction of RBP-4 in alcoholic cirrhosis reported by Kalafateli 2015 [26] and supported by the wider cirrhotic literature [20,21,31] reflects the predominant hepatocyte origin of circulating RBP-4 and progressive loss of synthetic capacity, modulated by renal clearance and vitamin A status. The seemingly contradictory finding of Chen 2025 [25], in which elevated baseline RBP-4 predicted incident MetALD/ALD, is fully consistent with this model: in pre-cirrhotic metabolic-adipose disease, RBP-4 functions as a classical adipokine signalling insulin resistance and ectopic lipid loading and is therefore expected to rise; once hepatocellular function deteriorates, the synthetic-failure signal supervenes, and RBP-4 falls. Chemerin, which is highly expressed by hepatocytes [17] and falls with worsening Child-Pugh stage in Prystupa 2019 [27] and in independent non-ALD cohorts [18,19], fits the same axis. The chemerin signal is particularly informative because Horn and colleagues showed that low circulating chemerin independently predicted 28-day mortality or need for liver transplantation in decompensated cirrhosis after adjustment for MELD, systemic inflammation and infection [19], indicating that with appropriate statistical adjustment the source-failure signal carries prognostic information independent of generic inflammatory confounders.

The second axis is impaired hepatic clearance and portal-systemic shunting. Omentin-1 occupies this limb. Eisinger and colleagues directly measured omentin-1 in paired portal, hepatic and systemic venous samples and demonstrated a portal vein > systemic vein > hepatic vein gradient indicative of hepatic extraction [22]. The roughly nine-fold elevation of plasma omentin-1 in alcoholic cirrhosis observed in Waluga 2019 [28], far exceeding values in NAFLD and in primary biliary cholangitis, is therefore more consistent with a portal-systemic axis marker than with a primary readout of adipose-tissue biology. This interpretation is reinforced by the contrast with the wider metabolic literature, in which omentin-1 typically falls in obesity and insulin resistance; the markedly elevated values in alcohol-related cirrhosis cannot plausibly be attributed to healthier adipose-tissue function and are most parsimoniously explained by failure of first-pass hepatic clearance and by portal-systemic shunting. The absence of correlation with Child-Pugh or MELD scores within the small alcoholic cirrhosis subgroup (*n* = 30) does not contradict this model; both scores aggregate synthetic, excretory and haemodynamic components, while omentin-1 specifically tracks the shunting axis, which is only partly reflected in those composite indices.

The third axis is the alcohol-exposure and inflammatory adipose-liver axis. eNAMPT is the prototype. Zhou and colleagues demonstrated that (i) serum eNAMPT is elevated in patients with ALD and correlates with indices of liver injury; (ii) ethanol selectively stimulates eNAMPT secretion from brown adipocytes-not from white adipocytes; (iii) brown-adipose-tissue-derived eNAMPT induces hepatocyte ferroptosis through TLR4-dependent mitochondrial reactive oxygen species generation and ferritinophagy; (iv) brown-adipose-tissue-specific Nampt knockdown attenuates alcoholic liver injury in vivo; and (v) antibody neutralisation of eNAMPT rescues the phenotype [23]. In this axis, the adipokine is not a passive correlate of disease severity but a candidate effector of brown-adipose-tissue-liver communication and a tractable therapeutic target. NAMPT/PBEF in its extracellular form has also long been recognised as an acute-phase inflammatory mediator [32], which is an additional reason to expect its concentration to rise in alcohol-associated hepatitis and acute-on-chronic liver failure. Vaspin remains a candidate non-specific or compensatory marker without a clear axis assignment on present data.

Two consequences follow. First, in advanced liver disease, adipokines cease to be a single diagnostic panel and become a phase-dependent readout of three biologies: loss of hepatocyte synthesis, impaired hepatobiliary-portal clearance, and alcohol-driven inflammatory dysfunction of the adipose-liver axis. Second, a biomarker of risk, a biomarker of phase, and a mechanistic mediator are not interchangeable categories. Chen 2025 [25] suggests that RBP-4 may operate as a risk biomarker in early metabolic-steatotic disease, whereas Kalafateli 2015 [26] and the supportive non-ALD literature [21,31] show that in established cirrhosis the same molecule reads out as a phase biomarker of failing synthetic reserve. Omentin-1 in cirrhosis does not behave as a classical metabolic adipokine but as a marker of altered portal-systemic circulation. eNAMPT, in light of Zhou 2024 [23], is more credibly a mechanistic mediator than a passive biomarker.

This framework is hypothesis-generating rather than directly demonstrated longitudinally. The trajectory from early metabolic-adipose disease through alcohol-exposed MetALD into cirrhotic source failure and, in a subset, post-abstinence recompensation, has not been characterised in a single, prospectively followed cohort with serial adipokine sampling. Longitudinal studies tracking these three axes across well-phenotyped MetALD and ALD cohorts, ideally with paired non-invasive fibrosis staging and verified alcohol-exposure data, are required to confirm or refute this model.

### 4.2. eNAMPT, Brown Adipose Tissue and a Paradigm Shift for Visfatin in ALD

The visfatin/NAMPT axis warrants particular attention because of a 2024 paradigm shift in mechanistic understanding [23]. It should be stated explicitly at the outset that this section rests on external, largely experimental mechanistic evidence rather than on any eligible clinical adipokine study included in the present systematic review: no included study measured eNAMPT with an isoform-specific assay in an ALD or MetALD population, and the single eligible study to report visfatin at all (Kalafateli 2015 [26]) did not specify the isoform measured and found no significant difference across disease stages. The material that follows is therefore mechanistic context that motivates future work, not a synthesised clinical finding of this review, and the eNAMPT axis should accordingly be regarded as the least clinically substantiated of the three proposed axes. This work transforms the interpretation of any visfatin or NAMPT measurement in an ALD cohort: serum eNAMPT in ALD is not a generic adipose-tissue marker but a candidate effector molecule of brown-adipose-tissue-liver communication and a tractable therapeutic target. Two corollaries follow for our review. First, the historical visfatin literature is not simply sparse but largely uninterpretable in modern terms: the indeterminate visfatin data from Kalafateli 2015 [26] cannot be re-interpreted because the isoform measured was not specified; assays available at the time did not reliably discriminate iNAMPT from eNAMPT, and the absolute concentrations obtained from different commercial kits are non-equivalent [33,34]. Second, future studies in ALD and MetALD should measure eNAMPT specifically using full-length-specific assays and consider standardised sampling for diurnal variation [35].

### 4.3. Mechanistic Links Between Alcohol, Adipose Tissue and Adipokine Biology

The mechanistic literature provides strong biological plausibility for treating adipokines in ALD as more than passive correlates of disease severity. Chronic ethanol exposure activates adipose triglyceride lipase and hormone-sensitive lipase, increases adipose-tissue free fatty acid efflux, and drives reverse triglyceride transport from adipose to liver [12,36], and the comprehensive synthesis by Parker and colleagues documents that chronic alcohol elevates circulating leptin, visfatin and chemerin while exacerbating pro-inflammatory and pro-fibrotic responses [12]. Hepatocyte-produced prochemerin protects against experimental steatohepatitis by deactivating peripheral blood mononuclear cells and reducing pro-inflammatory mediators, supporting a hepatoprotective role for hepatic chemerin and reinforcing the interpretation that falling serum chemerin in cirrhosis reflects loss of a protective hepatocyte-derived pool [17,37].

### 4.4. Why the Included Adipokines May Be Imperfect Biomarkers in ALD Specifically

Several confounders are under-addressed in the primary literature and deserve explicit acknowledgement. First, malnutrition, sarcopenia and “alcoholic adipopenia” alter the absolute concentration of every adipokine independently of hepatic biology. Skeletal muscle area declines at approximately −5.7% per year in ALD cirrhosis versus −2.8% in HBV and −3.1% in HCV cirrhosis, with ALD cirrhosis independently associated with rapid muscle loss and mortality (hazard ratio 2.43, 95% CI 1.12–5.28) [13]; subcutaneous adipose tissue is itself an independent mortality predictor in cirrhosis. Loss of the adipose secretory compartment in late disease will therefore lower circulating adipokine concentrations independently of hepatic clearance changes-a second, additive mechanism contributing to the phase-dependent trajectory described in Section 4.1.

Second, active drinking versus abstinence at the time of sampling almost certainly drives substantial between-study heterogeneity. None of the five included studies systematically reported time since last drink or used an objective alcohol biomarker. In presumed non-alcoholic fatty liver populations, hair ethyl glucuronide detects harmful alcohol consumption in approximately 29% of patients [9], indicating the magnitude of misclassification is likely also present in the older “alcoholic cirrhosis” cohorts. Critically, a single timepoint sample cannot resolve binge versus daily drinking, recent abstinence, or lifetime exposure, all of which modulate adipose-liver biology differently.

Third, assay heterogeneity is particularly severe. For visfatin/NAMPT, different commercial ELISAs target different epitopes and yield non-equivalent absolute concentrations; full-length-specific assays did not exist when several of the included studies were conducted; diurnal variation is well established; and only a minority of clinical studies distinguish biologically inactive iNAMPT from circulating eNAMPT-even though only the latter is the brown-adipose-tissue-derived effector implicated in alcoholic ferroptosis [23,33,34,35]. For chemerin, available ELISAs typically do not discriminate between proform and bioactive C-terminal isoforms, although the receptor biology (CMKLR1, GPR1, CCRL2) is highly isoform-dependent. For RBP-4, holo-RBP4, apo-RBP4, and truncated forms circulate in different proportions across hepatic and renal disease states and are not separately reported by standard immunoassays. For omentin-1 and vaspin, baseline directionality depends on the metabolic phenotype of the comparator: omentin-1 typically falls and vaspin typically rises in obesity and insulin resistance, so the same absolute concentration carries different biological meaning depending on body composition.

Fourth, inflammation is a generic confounder. Chemerin is upregulated by lipopolysaccharide and inflammatory cytokines, and NAMPT/PBEF is a canonical acute-phase mediator [32]. In decompensated ALD with bacterial translocation and acute-on-chronic liver failure, this inflammatory signal will overlay any adipose-specific signal. Encouragingly, Horn 2018 [19] showed that chemerin retained independent prognostic value after adjustment for systemic inflammation, infection and extrahepatic organ failure, suggesting that with appropriate statistical adjustment the adipokine signal can be isolated.

Fifth, renal clearance is critical for RBP-4. RBP-4 is more accurately conceptualised not as a pure marker of hepatic synthetic function but as the readout of a three-component hepatic synthetic-retinoid transport-renal clearance axis. It is synthesised predominantly by hepatocytes, circulates bound to retinol and transthyretin as a ternary complex, and is cleared by the kidney; its serum concentration therefore integrates hepatic synthetic reserve, vitamin A status and the size of the retinol-transthyretin binding pool, and renal function. In ALD and MetALD each of these limbs is perturbed independently: malnutrition and cholestasis disturb vitamin A handling and transthyretin (itself a negative acute-phase and nutritional marker), while diabetic nephropathy is prevalent in the metabolic-dysfunction component of MetALD. Chronic kidney disease elevates RBP-4 approximately 3-fold while chronic liver disease depresses it [21], and the two compartments are coupled in hepatorenal physiology and in MetALD where diabetic nephropathy is prevalent. Because a low serum RBP-4 in an ALD patient could reflect hepatic synthetic failure, vitamin A depletion or nutritional transthyretin loss, and a normal or high value could reflect coexisting renal impairment masking hepatic loss, future ALD/MetALD adipokine studies should routinely report estimated glomerular filtration rate or creatinine, nutritional indices, and, where feasible, vitamin A-related parameters (serum retinol and transthyretin) alongside RBP-4, so that the hepatic, retinoid-nutritional and renal contributions to the measured concentration can be disentangled.

Sixth, pre-analytical variables-diurnal variation, fasting status, freeze-thaw cycles-are inadequately reported in the original studies and represent additional methodological caveats.

### 4.5. The Hidden MetALD Problem and the Limits of Inference from Older Literature

None of the five included studies were designed according to the 2023 Rinella/AASLD-EASL-ALEH framework [4], and only Chen 2025 [25] uses the MetALD category. The “alcoholic cirrhosis” cohorts of Kalafateli 2015 [26], Prystupa 2019 [27] and Waluga 2019 [28] inevitably include patients with concomitant cardiometabolic dysfunction who would today be classified as MetALD-cirrhosis. Conversely, our review captures only the literature already explicitly labelled as ALD or MetALD and does not encompass the much larger body of historical NAFLD/MAFLD studies in which patients may now meet MetALD criteria, because alcohol thresholds in that literature were heterogeneous, mostly self-reported, and rarely verified with biomarkers. The 2025 expert panel position statement [7] emphasises that heavy alcohol use itself can produce hypertension, hypertriglyceridaemia and hyperglycaemia, so the application of a single cardiometabolic criterion can overdiagnose the metabolic component of MetALD; in the GALAXY cohort of individuals with a history of excessive alcohol intake, 98% had at least one cardiometabolic risk factor [5]. Contemporary trial-design proposals therefore advocate simultaneous evidence of at least two cardiometabolic features plus verified, quantifiable alcohol exposure recorded over the preceding 3–6 months [38]. Adding phosphatidylethanol to self-reported alcohol intake has been shown to quadruple MetALD diagnoses and triple ALD diagnoses [8], and to redistribute substantial proportions of presumed MASLD into MetALD or ALD in real-world cohorts [39].

The alcohol-metabolic interaction is supra-additive. Low-to-moderate alcohol consumption in MASLD interacts with cardiometabolic risk factors in a dose-dependent supra-additive manner to increase the risk of significant fibrosis [14], and moderate alcohol consumption synergises with type 2 diabetes mellitus to produce advanced fibrosis even in cohorts traditionally classified as non-alcoholic, with PEth values of at least 50 ng/mL marking a measurable inflexion in fibrosis risk [15]. The implication is that retrospective reclassification of older “NAFLD” or “alcoholic cirrhosis” series into modern MetALD categories is methodologically fragile, and any future cross-tabulation of adipokines against SLD subtype must prospectively apply the 2023 criteria and verify alcohol exposure with an objective biomarker [7,8,9].

A third nomenclature-related caveat concerns the absence of a contemporaneous reference comparator. None of the included studies compares circulating adipokines in ALD directly with a MASLD or MetALD cohort defined under the 2023 framework, and the broader adipokine literature in steatotic liver disease was generated under the legacy “NAFLD” or “MAFLD” definitions. NAFLD was an exclusion-based diagnosis that required the absence of significant alcohol consumption, competing aetiologies and chronic steatogenic medications, but used heterogeneous and largely self-reported alcohol cut-offs, typically 20–30 g/day for men and 10–20 g/day for women. MAFLD replaced this exclusion logic with positive metabolic criteria and explicitly allowed concomitant alcohol consumption, producing systematic non-overlap with both NAFLD and MASLD when the three definitions were applied to the same population. MASLD reinstated an alcohol exclusion threshold of at most 140 g/week for women and at most 210 g/week for men, with MetALD spanning the intermediate 140–350 g/week and 210–420 g/week windows. Adipokine reference values, cut-offs, and effect sizes derived under any of these earlier definitions, therefore, cannot be directly transposed to the post-2023 MASLD/MetALD/ALD framework, and pooled estimates spanning the nomenclature transition risk combining biologically non-comparable populations. The hair ethyl glucuronide data of Staufer 2022 [9] further imply that a substantial proportion of the historical “NAFLD adipokine” signal may have been generated in patients with undisclosed alcohol exposure who would today be reclassified as MetALD or ALD, so that the older comparator literature is not only nominally non-equivalent but biologically contaminated by misclassified alcohol use.

A fourth point deserves explicit emphasis: the destigmatising shift from “alcoholic” to “alcohol-related” and “alcohol-associated” terminology is not merely cosmetic. Older “alcoholic cirrhosis” cohorts, including all four pre-2023 included studies, were recruited at a time when the diagnostic label itself carried clinical stigma and was most often applied to patients presenting with advanced, often decompensated, disease. The phenotype captured in those cohorts is therefore biassed towards late-stage hospital-based ALD and is not representative of the broader contemporary ALD/MetALD spectrum, which extends from compensated steatotic liver disease in primary-care populations through to acute-on-chronic liver failure. Adipokine signals derived from late-stage, hospital-based, historically labelled “alcoholic” cirrhotics should therefore be generalised with caution to the broader post-2023 ALD/MetALD population.

### 4.6. Cirrhosis Is Not a Single State: Compensated, Decompensated, AH/ACLF and Recompensated Phenotypes

A second source of biological heterogeneity that is under-addressed in the included primary literature is that “alcoholic cirrhosis” encompasses several biologically distinct states. The Baveno VII framework has reframed the natural history of advanced chronic liver disease around compensated advanced chronic liver disease (cACLD), clinically significant portal hypertension (CSPH), hepatic decompensation, and hepatic recompensation following aetiological cure. In a recent multicentre study of decompensated alcohol-related cirrhosis, 33.8% of patients achieved Baveno VII recompensation by 5 years when sustained abstinence was maintained, with a substantial survival benefit and a negligible residual risk of liver-related death and hepatocellular carcinoma [40]. Earlier work has further linked alcohol abstinence to falls in hepatic venous pressure gradient and to clinically significant regression of portal hypertension [41].

These observations have direct implications for adipokine interpretation. cACLD, decompensated cirrhosis, acute-on-chronic liver failure (ACLF), and abstinence-induced recompensation differ in the integrity of hepatocyte synthetic function, in the magnitude of portal-systemic shunting, and in the burden of systemic inflammation and bacterial translocation. A single adipokine sampled at a single time point cannot reliably distinguish between a patient with biologically active alcoholic injury, a stably abstinent and recompensing patient, and an inflamed acute-on-chronic patient. None of the included cross-sectional studies stratified patients by recompensation status or by time since last drink; this is not a minor limitation because the three-axis framework explicitly predicts that adipokine signals will reverse direction or change axis dominance between these states.

A related phenotypic gap deserves explicit mention. The included studies focused on chronic alcohol-related cirrhosis. Alcohol-associated hepatitis (AH) and ACLF, both integral parts of the ALD spectrum, are essentially absent from the present evidence base. Because eNAMPT, chemerin, and several other candidate adipokines are also acute-phase or inflammation-responsive proteins, severe inflammatory or infectious overlay in AH and ACLF is expected to dominate the circulating adipokine signal in ways that are not captured by chronic stable cirrhosis cohorts. An honest reading of the present synthesis is therefore not “adipokines in ALD” but “adipokines in chronic alcohol-related cirrhosis and in the population-level risk of incident MetALD/ALD”.

### 4.7. Aetiology Versus Phase: A Conceptual Caveat

A further point closely connected to Section 4.6 is that, in advanced cirrhosis, the correlations of each adipokine with Child-Pugh, MELD, INR, bilirubin, and albumin are partly self-referential rather than aetiology-specific. These composite scores aggregate synthetic, excretory and circulatory failure, so an adipokine that tracks them reliably is, in effect, reporting the global hepatic failure phenotype rather than the specific biology of alcohol. This is particularly relevant for RBP-4 and chemerin, both of which fall in advanced cirrhosis irrespective of underlying aetiology and both of which are subject to additional modulation by renal function, vitamin A status and inflammation [20,21]. The present synthesis therefore supports a careful framing in which adipokines in ALD cirrhosis are interpreted primarily as reporters of disease phase and axis dominance, not as aetiology-specific signals; aetiological specificity, if it exists, is most plausibly expected for the alcohol-exposure axis (eNAMPT) and is not yet established for the source-failure axis.

### 4.8. Diagnostic Utility Versus Biological Interpretability

A practical distinction emerging from this synthesis is that biological interpretability and diagnostic utility are not equivalent and should not be conflated. The reported diagnostic AUC for chemerin in alcoholic cirrhosis was non-significant (0.653 in Prystupa 2019) [27], the prospective AUC for RBP-4 in predicting incident MetALD/ALD was only modest (0.672 in males in Chen 2025) [25], and no ALD-specific diagnostic AUC is available for omentin-1, vaspin, or visfatin. None of the included data therefore supports the use of any single adipokine as a ready-made diagnostic tool capable of discriminating ALD from MetALD, or alcohol-related cirrhosis from other cirrhoses. What the data do support is the use of these adipokines as biologically interpretable reporters of which axis is dominant in a given patient at a given moment, particularly when combined with established non-invasive tests such as FIB-4, the Enhanced Liver Fibrosis test and vibration-controlled transient elastography, and with an objective alcohol biomarker such as PEth. In a recent health check-up cohort, the diagnostic performance of FIB-4 for advanced fibrosis was similar in MetALD and in MASLD [42], supporting its role as a robust initial screening tool. Adipokines should therefore not be positioned as replacements for FIB-4, ELF or transient elastography but as orthogonal carriers of biology that classical fibrosis-oriented tests do not capture: the adipose-portal-hepatic triangle.

### 4.9. Population and External Validity

The five included studies span four distinct populations: rural-suburban China (Chen 2025) [25], urban Greece (Kalafateli 2015) [26], urban Poland (Prystupa 2019 and Waluga 2019) [27,28], and South Korea (Kwon 2009) [29]. Three population-level considerations deserve emphasis. (i) Drinking patterns: Poland is characterised by spirits-dominant consumption with a documented rise in alcohol-related mortality across 2002–2017 following the relaxation of alcohol-control measures, producing pharmacokinetic, gut-microbiota and adipose-tissue exposure profiles distinct from wine-dominant Mediterranean cohorts. Adipokine cut-offs derived in one drinking culture are unlikely to transfer cleanly to another. (ii) Sex distribution: the contemporary alcohol-associated cirrhosis burden has shifted disproportionately to young women [3], yet our included cohorts are male-skewed (Kalafateli 2015 [26]: 87.5% male; Polish cohorts likewise male-predominant). Because omentin and chemerin both vary by visceral-adipose-tissue distribution and because oestrogen modulates RBP-4 metabolism, male-derived reference ranges may misclassify female patients. (iii) Age: alcoholic cirrhotics are systematically younger than viral or cryptogenic cirrhotics; younger patients have different adipose-tissue composition and hormonal milieu, so age-stratified reference ranges will be required for clinical translation.

### 4.10. Search-Strategy and Methodological Limitations

Several sources of retrieval bias must be acknowledged. First, disease-term evolution and nomenclature transition: searches not explicitly OR-ing the destigmatising shift from “alcoholic” to “alcohol-related” and “alcohol-associated” liver disease, together with the successive steatotic liver disease definitions “NAFLD”, “MAFLD”, “MASLD” and “MetALD”, will under-recover relevant literature. The four steatotic liver disease definitions further use different alcohol thresholds (NAFLD: variable, commonly 20–30 g/day for men and 10–20 g/day for women; MAFLD: concomitant alcohol permitted; MASLD: at most 140 and 210 g/week for women and men; MetALD: 140–350 and 210–420 g/week) and different metabolic criteria, such that a single search string cannot retrieve a biologically homogeneous population across the transition and pooled inferences spanning the nomenclature change must be treated as exploratory. Second, molecule nomenclature: visfatin, NAMPT and PBEF1 are interchangeable names for one protein, but eNAMPT and iNAMPT are biologically distinct and are not separately MeSH-indexed; chemerin is also indexed as RARRES2 or TIG2, and omentin-1 as ITLN1. Third, our 50-ALD-aetiology threshold for inclusion of mixed-aetiology cohorts is conventional but arbitrary. Fourth, grey literature, conference proceedings, preprints and non-English papers may have been substantially under-represented. We did not systematically search the proceedings of major hepatology congresses (EASL International Liver Congress, AASLD The Liver Meeting, APASL Annual Meeting), preprint servers (medRxiv, bioRxiv), trial and protocol registries beyond ClinicalTrials.gov, theses, or institutional repositories. This is non-trivial for the present topic: adipokine measurements in small single-centre hepatology cohorts are well-suited to poster and oral abstracts that frequently never proceed to full publication, particularly when results are null or directionally inconsistent. The absence of formal grey-literature retrieval therefore probably introduces a directional bias favouring statistically significant and “positive” findings (publication bias), and the summary effect sizes reported here should be regarded as upper-bound rather than unbiased estimates. With only five included studies and at most three per analyte, formal small-study-effect or publication-bias testing (funnel plot, Egger test, trim-and-fill) was not feasible, so this bias cannot be quantified within the present dataset. In addition, native-language bibliographic indices for major non-English literatures (eLibrary.ru for Russian, CNKI for Mandarin, SciELO for Spanish and Portuguese, Polska Bibliografia Lekarska for Polish) were not interrogated; Kwon 2009 [29] is a Korean-language paper indexed in PubMed via translated abstract and represents the only paper retrieved in the non-English-language category. Fifth, the use of the Newcastle-Ottawa Scale is acknowledged as suboptimal for purely cross-sectional designs; three of our five included studies are cross-sectional and may carry residual unmeasured bias from reverse causality, end-stage case selection and biomarker-related verification that NOS does not fully capture. Sixth, our review is fundamentally limited by the very small number of eligible studies per analyte, precluding meta-analysis, and by the marked methodological heterogeneity described above.

The conflict of interest in Waluga 2019 [28], in which one co-author of the present review (M. Kukla) is also a co-author of that study, was managed by independent data extraction performed by K.M. and B.B-S.; this study is identified explicitly in Table 3 and in the Conflicts of Interest Section.

### 4.11. Future Directions

Three concrete clinical roles for adipokines in ALD and MetALD deserve prospective testing. First, distinguishing MetALD from pure ALD: this is currently the most pressing pathology-clinical challenge in steatotic liver disease, and adipokines-particularly a ratio of metabolic-dysfunction-driven analytes (RBP-4, total chemerin) to alcohol-driven analytes (eNAMPT specifically)-could in principle complement objective alcohol biomarkers and cardiometabolic profiling. No study to date has prospectively evaluated this proposition. Second, integration with non-invasive tests: adipokines should not be proposed as replacements for FIB-4, ELF, or transient elastography, but as adjuncts capturing orthogonal biology (the adipose-portal-hepatic axis that hepatocellular-injury markers do not measure). Third, druggability: A1120 and related non-retinoid RBP-4 antagonists, CMKLR1/GPR1 antagonists for chemerin signalling, recombinant omentin-1 for endothelial dysfunction, and eNAMPT-neutralising antibody (which attenuates alcoholic liver injury in mice) [23] are all preclinically tractable and have not been tested in ALD or MetALD trials.

We propose that a definitive prospective MetALD-versus-pure-ALD-versus-MASLD study should include: (i) standardised adipokine-isoform measurement (full-length eNAMPT, isoform-specific chemerin); (ii) verified alcohol-exposure stratification using PEth for recent (2–4 weeks) exposure complemented by hair ethyl glucuronide for longer (up to 3 months) windows [8,9,38]; (iii) inclusion criteria requiring at least two cardiometabolic features plus quantifiable alcohol exposure over the preceding 3–6 months, as advocated for contemporary MetALD trials [38]; (iv) protocolised sampling controlling for diurnal and fasting effects; (v) integration with FIB-4, ELF, transient elastography and clinical scores (Maddrey discriminant function, MELD, ABIC) in multivariable risk models; (vi) explicit stratification by Baveno VII phenotype, including cACLD, decompensated cirrhosis, ACLF and post-abstinence recompensation [40]; and (vii) longitudinal sampling across abstinence intervention to test the hypothesis that eNAMPT and chemerin trajectories track histological resolution. Collaboration with international consortia such as Global AlcHep may facilitate the recruitment of sufficiently large and well-characterised cohorts.

Beyond measuring adipokines as isolated circulating biomarkers, future ALD and MetALD studies would benefit from integrating them with pharmacodynamic and mechanistic read-outs of the adipose-liver and gut-liver axes. The pathophysiology of ALD is tightly coupled to intestinal dysbiosis and inflammasome activation on the one hand [43] and to lipid peroxidation and oxidative stress on the other [44], both of which plausibly modulate adipose-tissue secretory function and hepatic adipokine handling. Co-measuring isoform-specific adipokines alongside lipidomic and metabolomic panels, gut-liver-axis and bacterial-translocation markers, oxidative- and endoplasmic-reticulum-stress and ferroptosis-related read-outs, and non-invasive fibrosis assessment could therefore help to resolve which of the three proposed axes dominates in a given patient, and to connect the adipokine signal to the underlying inflammatory and lipid-peroxidative biology of alcohol-related injury. Such multi-omics integration is a natural extension of the framework proposed here and a priority for translational validation.

## 5. Conclusions

Across a small but conceptually rich evidence base, RBP-4 and chemerin decrease in alcohol-related cirrhosis in proportion to the loss of hepatic synthetic function, while omentin-1 is paradoxically elevated, most plausibly reflecting impaired hepatic catabolism and portal-systemic shunting. Elevated baseline RBP-4 independently predicts incident MetALD/ALD over five years in a prospective community cohort. It should be emphasised that these observations derive almost entirely from chronic alcohol-related cirrhosis, supplemented by a single population cohort providing limited incident MetALD/ALD risk data; they therefore characterise the advanced and the pre-clinical extremes of the disease, rather than the full ALD/MetALD spectrum, and the directly informative alcohol-related cirrhosis signal rests on roughly 169 cases across three cross-sectional cohorts. Vaspin shows no consistent signal, and visfatin/NAMPT data are largely uninterpretable, even though the recent identification of brown-adipose-tissue-derived eNAMPT as a TLR4-dependent driver of alcoholic ferroptosis renders this axis the single most promising adipokine candidate for further investigation in ALD. The available data are best interpreted not as a single diagnostic panel but as a phase-dependent readout of three biological axes: hepatic source failure, impaired hepatic clearance with portal-systemic shunting, and the alcohol-exposure and inflammatory adipose-liver axis. This framework is hypothesis-generating rather than longitudinally demonstrated. The evidence base remains too sparse and too heterogeneous to support clinical application or quantitative synthesis, and primary prospective studies using standardised, isoform-specific assays, objective alcohol biomarkers, and the post-2023 SLD/MetALD nomenclature, stratified by Baveno VII phenotype, are urgently needed.

## Figures and Tables

**Table 1:** Characteristics of included studies.

Study	Design	Country	Population	Adipokine(s)	Key Findings	NOS
Kalafateli 2015 [26]	Prospective cross-sectional	Greece	ALC *n* = 40, non-diabetic, abstinent for at least 6 months, no controls	Visfatin, RBP-4	RBP-4 decreases with Child-Pugh stage (*p* = 0.006); inversely correlated with MELD (r = −0.439)	7-Low
Chen 2025 [25]	Prospective cohort (5-year)	China	Community cohort n = 3504; 67 incident MetALD/ALD	RBP-4	Elevated baseline RBP-4 independently predicts MetALD/ALD (RR per-SD 1.64, 95% CI 1.29–2.08)	9-Low
Prystupa 2019 [27]	Cross-sectional	Poland	ALC *n* = 99 (Child A/B/C: 29/36/34); controls *n* = 20	Chemerin	Chemerin decreases with cirrhosis stage; controls vs. Child C *p* = 0.01; AUC = 0.653 (ns)	6-Moderate
Waluga 2019 [28]	Cross-sectional	Poland	NAFLD *n* = 25, PBC *n* = 20, AC *n* = 30; controls *n* = 25	Omentin-1, Vaspin	Omentin highest in AC vs. all groups (*p* < 0.01); vaspin: no significant differences	5-Moderate
Kwon 2009 [29] (contextual)	Retrospective	Republic of Korea	Mixed-aetiology CLD *n* = 573 (alcohol 9.0%, not separated); controls *n* = 40	RBP-4	RBP-4 declines with CLD severity (*p* < 0.001); AUC = 0.856 for Child B/C cirrhosis. Not ALD-specific.	4-High

AC, alcohol-related cirrhosis (retained from the original study, which used the historical term “alcoholic cirrhosis”); CLD, chronic liver disease; PBC, primary biliary cholangitis; NAFLD, non-alcoholic fatty liver disease; ns, not significant; NOS, Newcastle-Ottawa Scale.

**Table 2:** Summary of adipokine concentrations and key statistical findings.

Adipokine	Study	ALD Group (Mean ± SD or Median [IQR])	Control/Comparator	Key Comparison	Association with Disease Severity
RBP-4	Kalafateli 2015 [26]	CPA: 6.48 ± 3.2; CPB: 6.56 ± 3.37; CPC: 2.89 ± 2.07 microg/mL	No control group	Across CPA/B/C: *p* = 0.006	r = −0.439 vs. MELD; beta = −0.328 vs. Child-Pugh (multivariate)
RBP-4	Chen 2025 [25]	MetALD/ALD incid.: median 62.0 [49.5–73.0] mg/L	No-SLD: median 48.0 [39.0–60.0] mg/L	*p* < 0.001; RR per-SD 1.64 (1.29–2.08)	AUC = 0.672 (MetALD/ALD vs. no SLD)
RBP-4	Kwon 2009 [29] (contextual)	CH: 3.6 ± 2.0; Child A: 2.6 ± 1.6; Child B + C: 1.6 ± 1.0 mg/dL	Normal controls: 4.3 ± 1.1 mg/dL	All pairwise *p* < 0.001	AUC = 0.856 (Child B + C); r = 0.541 vs. PT
Chemerin	Prystupa 2019 [27]	CPA: 175.7 ± 62.7; CPB: 150.2 ± 59.7; CPC: 110.3 ± 73.6 ng/mL	Controls: 182.6 ± 80.4 ng/mL	Controls vs. CPC: *p* = 0.01	AUC = 0.653 (ns); r = −0.56 vs. INR
Omentin-1	Waluga 2019 [28]	AC: 1054.5 [579.7–2208.9] ng/mL	Controls: 114.5 [57.3–176.8] ng/mL	AC vs. all groups: *p* < 0.01	r = 0.502 vs. bilirubin; no correlation with MELD
Vaspin	Waluga 2019 [28]	AC: 0.27 [0.093–0.84] ng/mL	Controls: 0.26 [0.185–0.32] ng/mL	ns	No significant correlation with severity markers
Visfatin	Kalafateli 2015 [26]	CPA: 3.74; CPB: 12.32; CPC: 4.50 ng/mL (medians)	No control group	*p* = 0.536 (ns)	No significant trend after fat mass adjustment

CPA/B/C, Child-Pugh class A/B/C; AC, alcohol-related cirrhosis (retained from the original study, which used the historical term “alcoholic cirrhosis”); CH, chronic hepatitis; PT, prothrombin time; INR, international normalised ratio; MELD, Model for End-Stage Liver Disease; AUC, area under the ROC curve; ns, not significant; SD, standard deviation; IQR, interquartile range; RR, relative risk; per-SD, per standard deviation increment. Confounders adjusted: none of the three RBP-4 studies (Kalafateli 2015 [26], Chen 2025 [25], Kwon 2009 [29]) adjusted the reported RBP-4 concentrations or associations for renal function (estimated glomerular filtration rate or creatinine), despite the strong dependence of circulating RBP-4 on renal clearance; Chen 2025 [25] adjusted its incident-risk relative risks for 12 metabolic covariates but not for renal function, whereas Kalafateli 2015 [26] and Kwon 2009 [29] report unadjusted concentrations. The absence of renal adjustment should be borne in mind when interpreting all RBP-4 comparisons in this table (see Section 4.4).

**Table 3:** Newcastle-Ottawa Scale risk-of-bias assessment.

NOS Item	Kalafateli 2015 [26]	Chen 2025 [25]	Prystupa 2019 [27]	Waluga 2019 [28]	Kwon 2009 [29]	Max
SELECTION						**4**
1. Representativeness of exposed cohort	1	1	1	1	1	
2. Selection of non-exposed cohort	N/A	1	1	1	1	
3. Ascertainment of exposure	1	1	1	1	1	
4. Outcome not present at start	0	1	0	0	0	
COMPARABILITY						**2**
5a. Controlled for BMI	1	1	0	0	0	
5b. Controlled for diabetes	1	1	1	0	0	
OUTCOME						**3**
6. Validated assay	1	1	1	1	1	
7. Follow-up adequate	0	1	0	0	0	
8. Completeness of follow-up	1	1	1	1	0	
**TOTAL**	7	9	6	5	4	**9**
**Risk of bias**	**Low**	**Low**	**Moderate**	**Moderate**	**High**	

N/A, not applicable; NOS, Newcastle-Ottawa Scale.

## Data Availability

The original contributions presented in this study are included in the article/Supplementary Materials. Further inquiries can be directed to the corresponding author.

## Supplementary Materials

The following supporting information can be downloaded at: https://www.mdpi.com/article/10.3390/ijms27146509/s1.

## Abbreviations

The following abbreviations are used in this manuscript:

AC	alcoholic cirrhosis
ACLF	acute-on-chronic liver failure
AH	alcohol-associated hepatitis
ALD	alcohol-related liver disease
AUC	area under the ROC curve
cACLD	compensated advanced chronic liver disease
CSPH	clinically significant portal hypertension
ELF	Enhanced Liver Fibrosis test
eNAMPT	extracellular nicotinamide phosphoribosyltransferase
FIB-4	Fibrosis-4 index
HOMA-IR	homeostasis model assessment of insulin resistance
HVPG	hepatic venous pressure gradient
iNAMPT	intracellular nicotinamide phosphoribosyltransferase
MASLD	metabolic dysfunction-associated steatotic liver disease
MetALD	metabolic dysfunction and alcohol-related liver disease
NAFLD	non-alcoholic fatty liver disease
NAMPT	nicotinamide phosphoribosyltransferase
NOS	Newcastle-Ottawa Scale
PBC	primary biliary cholangitis
PEth	phosphatidylethanol
PRISMA	Preferred Reporting Items for Systematic Reviews and Meta-Analyses
RBP-4	retinol-binding protein 4
SLD	steatotic liver disease
SWiM	Synthesis Without Meta-Analysis
TLR4	Toll-like receptor 4
