# Supplementary material for: Circulating Adipokines in Alcohol-Related Liver Disease and MetALD: A Systematic Review and Structured Narrative Synthesis
Source: Int J Mol Sci. 2026 Jul 22;27(14):6509. doi: 10.3390/ijms27146509 (PMC13410293; doi:10.3390/ijms27146509)
Supplement: Supplementary file 1 [file ijms-27-06509-s001.zip › Supplementary File S1.pdf]

Supplementary File S1

Full electronic search strategy

Manuscript: Circulating adipokines in alcohol-related liver disease and MetALD: a systematic review and structured narrative synthesis (ijms-4414598).

Registration: PROSPERO CRD420261354251.

Databases, coverage and search date

Five electronic databases were searched from inception to 31 December 2025 (search date): PubMed/MEDLINE, Embase (Ovid), Web of Science Core Collection, Scopus and Cochrane CENTRAL. No language or publication-date restrictions were applied at the search stage. Reference lists of the included studies and of relevant reviews were additionally hand-searched.

Each search combined three concept blocks with the Boolean AND operator: (1) alcohol-related and chronic/steatotic liver disease terms; (2) the five target adipokines and their synonyms (chemerin, visfatin/NAMPT, vaspin, omentin-1 and RBP-4); and (3) alcohol-related exposure terms. The verbatim search strings executed in each database are reproduced below. Records retrieved were: PubMed/MEDLINE 56, Embase 64, Web of Science 103, Scopus 136 and Cochrane CENTRAL 14 (373 records in total; 258 duplicates removed, leaving 115 unique records for title and abstract screening).

1. PubMed / MEDLINE

```
(
  "alcoholic liver disease"[Title/Abstract] OR
  "alcohol-related liver disease"[Title/Abstract] OR
  "alcohol-associated liver disease"[Title/Abstract] OR
  "alcoholic hepatitis"[Title/Abstract] OR
  "alcoholic steatohepatitis"[Title/Abstract] OR
  "alcohol-associated steatohepatitis"[Title/Abstract] OR
  "alcoholic cirrhosis"[Title/Abstract] OR
  "alcohol-related cirrhosis"[Title/Abstract] OR
  "alcohol-associated cirrhosis"[Title/Abstract] OR
  "alcoholic steatosis"[Title/Abstract] OR
  "alcoholic fibrosis"[Title/Abstract] OR
  "liver cirrhosis"[Title/Abstract] OR
  "hepatic cirrhosis"[Title/Abstract] OR
  "chronic liver disease"[Title/Abstract] OR
  "liver fibrosis"[Title/Abstract] OR
  "MetALD"[Title/Abstract]
)
AND
(
  "chemerin"[Title/Abstract] OR
  "RARRES2"[Title/Abstract] OR
  "visfatin"[Title/Abstract] OR
  "NAMPT"[Title/Abstract] OR
  "PBEF"[Title/Abstract] OR
  "pre-B cell colony-enhancing factor"[Title/Abstract] OR
  "vaspin"[Title/Abstract] OR
  "SERPINA12"[Title/Abstract] OR
  "omentin"[Title/Abstract] OR
  "omentin-1"[Title/Abstract] OR
  "intelectin-1"[Title/Abstract] OR
  "ITLN1"[Title/Abstract] OR
  "retinol-binding protein 4"[Title/Abstract] OR
  "retinol binding protein 4"[Title/Abstract] OR
  "RBP-4"[Title/Abstract] OR
  "RBP4"[Title/Abstract]
)
AND
(
  "alcohol"[Title/Abstract] OR
  "alcoholic"[Title/Abstract] OR
  "alcohol-related"[Title/Abstract] OR
  "alcohol-associated"[Title/Abstract] OR
  "ethanol"[Title/Abstract]
)
```

Records retrieved: 56.

2. Embase (Ovid)

```
(
  'alcoholic liver disease':ab,ti OR
  'alcohol-related liver disease':ab,ti OR
  'alcohol-associated liver disease':ab,ti OR
  'alcoholic hepatitis':ab,ti OR
  'alcoholic steatohepatitis':ab,ti OR
  'alcohol-associated steatohepatitis':ab,ti OR
  'alcoholic cirrhosis':ab,ti OR
  'alcohol-related cirrhosis':ab,ti OR
  'alcohol-associated cirrhosis':ab,ti OR
  'alcoholic steatosis':ab,ti OR
  'alcoholic fibrosis':ab,ti OR
  'liver cirrhosis':ab,ti OR
  'hepatic cirrhosis':ab,ti OR
  'chronic liver disease':ab,ti OR
  'liver fibrosis':ab,ti OR
  'MetALD':ab,ti
)
AND
(
  'chemerin':ab,ti OR
  'RARRES2':ab,ti OR
  'visfatin':ab,ti OR
  'NAMPT':ab,ti OR
  'PBEF':ab,ti OR
  'pre-B cell colony-enhancing factor':ab,ti OR
  'vaspin':ab,ti OR
  'SERPINA12':ab,ti OR
  'omentin':ab,ti OR
  'omentin-1':ab,ti OR
  'intelectin-1':ab,ti OR
  'ITLN1':ab,ti OR
  'retinol-binding protein 4':ab,ti OR
  'retinol binding protein 4':ab,ti OR
  'RBP-4':ab,ti OR
  'RBP4':ab,ti
)
AND
(
  'alcohol':ab,ti OR
  'alcoholic':ab,ti OR
  'alcohol-related':ab,ti OR
  'alcohol-associated':ab,ti OR
  'ethanol':ab,ti
)
```
Records retrieved: 64.

3. Web of Science (Core Collection)

```
TS=(
  ("alcoholic liver disease" OR "alcohol-related liver disease" OR
   "alcohol-associated liver disease" OR "alcoholic hepatitis" OR
   "alcoholic steatohepatitis" OR "alcoholic cirrhosis" OR
   "alcohol-related cirrhosis" OR "alcoholic steatosis" OR
   "alcoholic fibrosis" OR "liver cirrhosis" OR
   "hepatic cirrhosis" OR "chronic liver disease" OR
   "liver fibrosis" OR "MetALD")
  AND
  ("chemerin" OR "RARRES2" OR "visfatin" OR "NAMPT" OR "PBEF" OR
   "vaspin" OR "SERPINA12" OR "omentin" OR "omentin-1" OR
   "intelectin-1" OR "ITLN1" OR "retinol-binding protein 4" OR
   "retinol binding protein 4" OR "RBP-4" OR "RBP4")
  AND
  ("alcohol" OR "alcoholic" OR "alcohol-related" OR
   "alcohol-associated" OR "ethanol")
)
```
Records retrieved: 103.

4. Scopus

```
TITLE-ABS-KEY(
  ("alcoholic liver disease" OR "alcohol-related liver disease" OR
   "alcohol-associated liver disease" OR "alcoholic hepatitis" OR
   "alcoholic steatohepatitis" OR "alcoholic cirrhosis" OR
   "alcohol-related cirrhosis" OR "alcoholic steatosis" OR
   "alcoholic fibrosis" OR "liver cirrhosis" OR
   "hepatic cirrhosis" OR "chronic liver disease" OR
   "liver fibrosis" OR "MetALD")
```

```
  AND
  ("chemerin" OR "RARRES2" OR "visfatin" OR "NAMPT" OR "PBEF" OR
   "vaspin" OR "SERPINA12" OR "omentin" OR "omentin-1" OR
   "intelectin-1" OR "ITLN1" OR "retinol-binding protein 4" OR
   "retinol binding protein 4" OR "RBP-4" OR "RBP4")
  AND
  ("alcohol" OR "alcoholic" OR "alcohol-related" OR
   "alcohol-associated" OR "ethanol")
)
```

Records retrieved: 136.

5. Cochrane CENTRAL

```
(
  "alcoholic liver disease" OR "alcohol-related liver disease" OR
  "alcohol-associated liver disease" OR "alcoholic hepatitis" OR
  "alcoholic cirrhosis" OR "liver cirrhosis" OR
  "chronic liver disease" OR "liver fibrosis" OR "MetALD"
):ti,ab,kw
AND
(
  "chemerin" OR "visfatin" OR "NAMPT" OR "vaspin" OR
  "omentin" OR "omentin-1" OR "intelectin-1" OR
  "retinol-binding protein 4" OR "RBP-4" OR "RBP4"
):ti,ab,kw
AND
(
  "alcohol" OR "alcoholic" OR "ethanol"
):ti,ab,kw
```

Records retrieved: 14.
