# Supplementary material for: Circulating Adipokines in Alcohol-Related Liver Disease and MetALD: A Systematic Review and Structured Narrative Synthesis
Source: Int J Mol Sci. 2026 Jul 22;27(14):6509. doi: 10.3390/ijms27146509 (PMC13410293; doi:10.3390/ijms27146509)
Supplement: Supplementary file 1 [file ijms-27-06509-s001.zip › Supplementary File S2.pdf]

International Journal of Molecular Sciences

MDPI

PRISMA 2020 Checklist

Manuscript: *Circulating adipokines in alcohol-related liver disease and MetALD: a systematic review and structured narrative synthesis*

Registration: PROSPERO CRD420261354251 (one minor amendment recorded after registration).

Section and Topic	Item #	Checklist item	Location where item is reported
**TITLE**			
**Title**	1	Identify the report as a systematic review.	Title (identified as "a systematic review and structured narrative synthesis").
**ABSTRACT**			
**Abstract**	2	See the PRISMA 2020 for abstracts checklist.	Abstract (single-paragraph summary stating objectives, information sources and search dates, eligibility, synthesis method [SWiM], number of included studies, principal findings and conclusions).
**INTRODUCTION**			
**Rationale**	3	Describe the rationale for the review in the context of existing knowledge.	Section 1 (Introduction), paragraphs 1-4.
**Objectives**	4	Provide an explicit statement of the objective(s) or question(s) the review addresses.	Section 1 (Introduction), final paragraph.
**METHODS**			
**Eligibility criteria**	5	Specify the inclusion and exclusion criteria for the review and how studies were grouped for the syntheses.	Section 4.2 (Eligibility Criteria); grouping by analyte in Section 4.6.
**Information sources**	6	Specify all databases, registers, websites, organisations, reference lists and other sources searched or consulted to identify studies. Specify the date when each source was last searched or consulted.	Section 4.3 (PubMed/MEDLINE, Embase, Web of Science, Scopus, Cochrane CENTRAL; reference-list handsearching; search date: 31 December 2025).
**Search strategy**	7	Present the full search strategies for all databases, registers and websites, including any filters and limits used.	Section 4.3; full PubMed search string in Supplementary File S1.

Section and Topic	Item #	Checklist item	Location where item is reported
**Selection process**	**8**	Specify the methods used to decide whether a study met the inclusion criteria of the review, including how many reviewers screened each record and each report retrieved, whether they worked independently, and if applicable, details of automation tools used in the process.	Section 4.4 (two reviewers [K.M., B.B.-S.] screened independently across two stages; disagreements resolved by discussion; no automation tools used for selection).
**Data collection process**	**9**	Specify the methods used to collect data from reports, including how many reviewers collected data from each report, whether they worked independently, any processes for obtaining or confirming data from study investigators, and if applicable, details of automation tools used in the process.	Section 4.4 (pre-specified extraction form; independent extraction by two reviewers; for mixed-aetiology cohorts only the ALD subgroup was extracted where separately available).
**Data items**	**10a**	List and define all outcomes for which data were sought. Specify whether all results that were compatible with each outcome domain in each study were sought (e.g., for all measures, time points, analyses), and if not, the methods used to decide which results to collect.	Section 4.4 (circulating serum/plasma concentrations of the five target adipokines and their associations with disease severity); Sections 2.3-2.7.
**Data items**	**10b**	List and define all other variables for which data were sought (e.g., participant and intervention characteristics, funding sources). Describe any assumptions made about any missing or unclear information.	Section 4.4 (design, country, sample size, age, sex, BMI, alcohol exposure, diabetes status, ALD diagnostic criteria, assay details).
**Risk of bias in studies**	**11**	Specify the methods used to assess risk of bias in the included studies, including details of the tool(s) used, how many reviewers assessed each study and whether they worked independently, and if applicable, details of automation tools used in the process.	Section 4.5 (Newcastle-Ottawa Scale; two reviewers independently; consensus); Section 2.2; Table 2.
**Effect measures**	**12**	Specify for each outcome the effect measure(s) (e.g., risk ratio, mean difference) used in the synthesis or presentation of results.	Section 4.6 and Sections 2.3-2.7 (direction and magnitude of between-group differences, correlation coefficients and diagnostic AUC; no pooled effect measure, as synthesis was narrative).
**Synthesis methods**	**13a**	Describe the processes used to decide which studies were eligible for each synthesis (e.g., tabulating the study intervention characteristics and comparing against the planned groups for each synthesis (item #5).	Section 4.6 (studies grouped by adipokine analyte).

Section and Topic	Item #	Checklist item	Location where item is reported
**Synthesis methods**	**13b**	Describe any methods required to prepare the data for presentation or synthesis, such as handling of missing summary statistics, or data conversions.	Section 4.6 (summary statistics reported as published; no data conversions performed).
**Synthesis methods**	**13c**	Describe any methods used to tabulate or visually display results of individual studies and syntheses.	Section 4.6; Tables 1-3; Figures 1-2.
**Synthesis methods**	**13d**	Describe any methods used to synthesise results and provide a rationale for the choice(s). If meta-analysis was performed, describe the model(s), method(s) to identify the presence and extent of statistical heterogeneity, and software package(s) used.	Section 4.6 (Synthesis Without Meta-Analysis [SWiM]; quantitative meta-analysis not performed because fewer than three studies were available per analyte).
**Synthesis methods**	**13e**	Describe any methods used to explore possible causes of heterogeneity among study results (e.g., subgroup analysis, meta-regression).	Section 4.6 and Section 3.10 (sources of heterogeneity appraised narratively; formal subgroup analysis and meta-regression not feasible).
**Synthesis methods**	**13f**	Describe any sensitivity analyses conducted to assess robustness of the synthesised results.	Not applicable - no quantitative synthesis was undertaken (Section 4.6).
**Reporting bias assessment**	**14**	Describe any methods used to assess risk of bias due to missing results in a synthesis (arising from reporting biases).	Section 3.10 (publication and grey-literature bias appraised narratively; formal funnel-plot, Egger and trim-and-fill testing not feasible with fewer than ten studies).
**Certainty assessment**	**15**	Describe any methods used to assess certainty (or confidence) in the body of evidence for an outcome.	Section 4.6 (formal GRADE rating not applied owing to the absence of pooled estimates; study-level risk of bias appraised with the Newcastle-Ottawa Scale [Section 4.5]; certainty discussed narratively in Sections 3.4-3.10).
**RESULTS**			
**Study selection**	**16a**	Describe the results of the search and selection process, from the number of records identified in the search to the number of studies included in the review, ideally using a flow diagram.	Section 2.1; Figure 1 (PRISMA 2020 flow diagram).

Section and Topic	Item #	Checklist item	Location where item is reported
**Study selection**	**16b**	Cite studies that might appear to meet the inclusion criteria, but which were excluded, and explain why they were excluded.	Sections 2.1 and 4.2 (Kwon 2009 retained as contextual evidence only, below the a priori 50% ALD threshold); full-text exclusions summarised in Figure 1.
**Study characteristics**	**17**	Cite each included study and present its characteristics.	Table 1; Section 2.1.
**Risk of bias in studies**	**18**	Present assessments of risk of bias for each included study.	Table 2; Section 2.2.
**Results of individual studies**	**19**	For all outcomes, present, for each study: (a) summary statistics for each group (where appropriate) and (b) an effect estimate and its precision (e.g., confidence/credible interval), ideally using structured tables or plots.	Tables 1 and 3; Sections 2.3-2.7.
**Results of syntheses**	**20a**	For each synthesis, briefly summarise the characteristics and risk of bias among contributing studies.	Sections 2.3-2.7; Table 2.
**Results of syntheses**	**20b**	Present results of all statistical syntheses conducted. If a meta-analysis was conducted, present, for each, the summary estimate and its precision (e.g., confidence/credible interval) and measures of statistical heterogeneity. If comparing groups, describe the direction of the effect.	Sections 2.3-2.7 (narrative per-analyte synthesis with direction of effect; no meta-analysis performed).
**Results of syntheses**	**20c**	Present results of all investigations of possible causes of heterogeneity among study results.	Section 3.10 (narrative).
**Results of syntheses**	**20d**	Present results of all sensitivity analyses conducted to assess the robustness of the synthesised results.	Not applicable-no quantitative synthesis (Section 4.6).
**Reporting biases**	**21**	Present assessments of risk of bias due to missing results (arising from reporting biases) for each synthesis assessed.	Section 3.10.
**Certainty of evidence**	**22**	Present assessments of certainty (or confidence) in the body of evidence for each outcome assessed.	Sections 3.4-3.10 and Section 5 (Conclusions) (narrative appraisal; formal GRADE rating not undertaken, see item 15).
**DISCUSSION**			
**Discussion**	**23a**	Provide a general interpretation of the results in the context of other evidence.	Section 3 (opening) and Section 3.1.
**Discussion**	**23b**	Discuss any limitations of the evidence included in the review.	Sections 3.4-3.9.
**Discussion**	**23c**	Discuss any limitations of the review processes used.	Section 3.10.

Section and Topic	Item #	Checklist item	Location where item is reported
**Discussion**	**23d**	Discuss implications of the results for practice, policy, and future research.	Section 3.11 (Future Directions); Section 5 (Conclusions).
**OTHER INFORMATION**			
**Registration and protocol**	**24a**	Provide registration information for the review, including the register name and registration number, or state that the review was not registered.	Section 4.1 (PROSPERO; registration number CRD420261354251).
**Registration and protocol**	**24b**	Indicate where the review protocol can be accessed, or state that a protocol was not prepared.	Section 4.1 (protocol accessible via the PROSPERO record CRD420261354251).
**Registration and protocol**	**24c**	Describe and explain any amendments to information provided at registration or in the protocol.	Section 4.1: one minor amendment to the registered protocol was made after registration and is documented in the PROSPERO record (CRD420261354251); all other methods were conducted as registered.
**Support**	**25**	Describe sources of financial or non-financial support for the review, and the role of the funders or sponsors in the review.	Funding ("This research received no external funding").
**Competing interests**	**26**	Declare any competing interests of review authors.	Conflicts of Interest (M. Kukla co-authored an included study [Waluga 2019]; data extraction was managed independently by K.M. and B.B.-S.).
**Availability of data, code and other materials**	**27**	Report which of the following are publicly available and where they can be found: template data collection forms; data extracted from included studies; data used for all analyses; analytic code; any other materials used in the review.	Data Availability Statement (extracted data available from the corresponding author on request); Supplementary File S1 (full search strategy); Supplementary File S2 (this completed PRISMA 2020 checklist).

From: Page MJ, McKenzie JE, Bossuyt PM, Boutron I, Hoffmann TC, Mulrow CD et al. The PRISMA 2020 statement: an updated guideline for reporting systematic reviews. BMJ 2021;372:n71. doi: 10.1136/bmj.n71. For more information, visit: http://www.prisma-statement.org/

Supplementary Table S1. JBI Critical Appraisal Checklist for Analytical Cross-Sectional Studies applied to the three cross-sectional studies included in the review.

JBI item (analytical cross-sectional studies)	Kalafateli 2015	Prystupa 2019	Waluga 2019
1. Were the criteria for inclusion in the sample clearly defined?	Yes	Yes	Yes
2. Were the study subjects and the setting described in detail?	Yes	Yes	Yes
3. Was the exposure measured in a valid and reliable way?	Yes	Yes	Yes
4. Were objective, standard criteria used for measurement of the condition?	Yes	Yes	Yes
5. Were confounding factors identified?	Yes (diabetes excluded)	Partial	Partial
6. Were strategies to deal with confounding factors stated?	Yes (non-diabetic; fat-mass adjustment)	No (no BMI/renal adjustment)	No (no BMI adjustment)
7. Were the outcomes measured in a valid and reliable way?	Yes (validated ELISA)	Yes (validated ELISA)	Yes (validated ELISA)
8. Was appropriate statistical analysis used?	Yes	Yes	Yes
**Overall appraisal**	**Include (low concern)**	**Include (moderate concern: confounding)**	**Include (moderate concern: confounding, small n)**

Y, Yes; N, No; U, Unclear; NA, not applicable. "Partial" indicates that confounders were named for some but not all analyses. Appraisal performed independently by K.M. and B.B.-S.; M. Kukla did not participate. Checklist source: Moola S, Munn Z, Tufanaru C, et al. Systematic reviews of etiology and risk. In: JBI Manual for Evidence Synthesis. JBI; 2020.
